# Pennsylvania Medical College

**Published:** 1861-09

**Authors:** 


PENNSYLVANIA MEDICAL COLLEGE.—The faculty of this institution have resigned in a body, owing to an unwillingness on the part of the Trustees to reduce their rent, in view of the hard times.

